# The training contents, problems and needs of doctors in urban community health service institutions in China

**DOI:** 10.1186/s12875-018-0867-6

**Published:** 2018-11-28

**Authors:** Shuang Shao, Tao Wu, Aimin Guo, Guanghui Jin, Rui Chen, Yali Zhao, Juan Du, Xiaoqin Lu

**Affiliations:** 1 School of General Practice and Continuing Education, Capital Medical University, Beijing, 100069 China; 2 Department of Scientific Research, Beijing Anzhen Hospital, Capital Medical University, Beijing, 100029 China

**Keywords:** Urban community health care service institutions, Doctors, Training programs, Training needs

## Abstract

**Background:**

The Chinese government offered various types of training programs for strengthening the role of doctors working in community health service institutions (CHSIs). The study intended to investigate the current training programs and training needs of doctors nationally in urban CHSIs in China, and to provide propositions for training more qualified doctors in the future.

**Methods:**

Total 3098 doctors in 192 urban CHSIs were chosen from 9 provinces (*Hebei, Liaoning, Shandong, Zhejiang, Fujian, Hunan, Guangxi, Guizhou, Ningxia*) and one municipality (*Beijing*) among 31 provinces in eastern, central, and western regions by stratified sampling methods in Mainland China. All doctors in the selected CHSIs were investigated in this study. We discharged 3073 questionnaires, and the response rate was 98.0%. Descriptive statistics were used to describe the characteristics, training contents, problems and needs of doctors. Differences in training contents, problems and needs between eastern, central and western regions were analyzed with chi-square tests.

**Results:**

49.3% of doctors in CHSIs had Bachelor’s degree and beyond. 12.9% of doctors had senior professional titles. The most frequent training topics for the doctors in eastern, central and western regions were “basic clinical theory knowledge” (52.4%), “community health service competency” (59.6%), “clinical practice skills” (45.9%) respectively. The most serious problem for doctors was “insufficient training time” in eastern (36.8%), central (36.5%) and western (39.6%). The biggest knowledge need for doctors both in eastern (79.8%) and central region (79.1%) was “the updated international medical knowledge”, in western region it was “the updated domestic medical knowledge” (73.2%). The biggest skill-related training need for doctors in eastern region (84.1%) and central region (82.6%) was “communication skills”, and “diagnosis and differential diagnosis” in western region (78.2%).

**Conclusion:**

Government should design proper training contents according to the knowledge and skill needs of different design. Furthermore, a uniform, rigorous training and evaluation system focus on practicability should be established to promote community health service system in Mainland China.

**Electronic supplementary material:**

The online version of this article (10.1186/s12875-018-0867-6) contains supplementary material, which is available to authorized users.

## Background

General practitioners (GPs, also called family physicians) are the first contact for patients within the health care system, providing preventive and medical services, referral to specialist and acute care when necessary, and playing a key role in community health service in China. There were 109,794 GPs which accounted for 5.6% of all registered physicians in 2012, and the number was far from the government’s goal of 300,000 GPs by 2020 [1, 2]. The goal is to reach the ratio of at least two or three GPs per 10,000 citizens by 2020, however, the current ratio is 1.07 [3]. The total number of outpatient visits increased from 2010 to 2013 at all health care facilities, and the outpatient rate of hospital increased by 34.4%, while the outpatient rate of community health service institutions (CHSIs) increased by only 19.7% [2]. The data concealed the fact that CHSIs were unable to attract a greater number of patients. Because of the lack of confidence in medical skills and the limited supply of advanced equipment and instruments in CHSIs, patients are not motivated to actively seek care in CHSIs for further treatments [4–7]. Thus, new and innovative initiatives are urgently in need to increase the quantity and quality of doctors to improve primary health care services.

Up to now, the Chinese government has proposed various approaches to reorganize hospital care and has put much emphasis on developing CHSIs. Since 2009, approximately 30% of government funds were invested in enhancing supply-side public infrastructure and training primary health care providers [8]. Furthermore, since 2000, the Chinese government offered various types of training programs for strengthening the role of all CHSIs providers, particularly for doctors (including GPs and physicians of other medical specialty who will be trained to be qualified GPs). The Chinese government has sought curriculum assistance from the World Organization of Family Doctors (WONCA) to improve the Chinese education, training and practice system. The education and training programs comprised undergraduate education, on-the-job training program or job-transfer training program, postgraduate residency training program, and continuing medical education (CME) programs. In most of medical schools in China, the “introduction to general practice” curriculum which is within 36 credit hours comprises of lecture sessions and practice sessions during the 5 years of undergraduate education: (1) the lecture sessions was designed to learn basic theories and concepts of general practice for students, including whole person care and holistic model; (2) the practice sessions was designed to acquire the preliminary impression of CHSIs for students. Currently, the job-transfer training program (replaced on-the-job training program from 2010) and the postgraduate residency training program (officially initiated from 2011) are the main two GPs training programs. On-the-job training program and transfer-job training program which offer a transitional solution for the shortage of GPs have a 1-year (full-time) training duration and target at retraining less trained CHSIs doctors who received 3 to 5 years of medical education after high school or other medical specialties to become a qualified GP. The programs were designed to promote clinical practical skills and theoretical knowledge through three phases: (1) academic curriculum, (2) Hospital-based clinical rotation, and (3) CHSIs-based training [9]. The postgraduate residency training program which lasts for 33 months (full-time) is mainly for graduates with 5 to 8 years post-high school medical education background, and includes two stages: (1) Hospital-based Clinical rotation and (2) CHSIs-based training [10]. Meanwhile, there are also other training programs as complements to training qualified GPs [11]. As CME programs (part-time), the train-the-trainer program, which emphasizes the training of qualified trainers, and the training programs hold by CHSIs, were considered supplements to teaching and training programs. Although there are various training programs, lack of knowledge on adequacy of contents, strengths, weaknesses of current training and future needs in China. Therefore, it is necessary to investigate the current training and future training needs of GPs nationally in china, however, no such study has been carried out to date.

## Methods

### Ethics statement

The research methods were performed in accordance with approved guidelines. Data were obtained from a 2011 cross-sectional survey, the Nationwide Investigation on Health Professionals of Community Health Service Institutions in Urban China. The study was undertaken as a part of a program sponsored by the Department of Medical Science, Technology and Education in China’s Ministry of Health (renamed National Health and Family Planning Commission of the People’s Republic of China in 2013, National Health Commission of the People’s Republic of China in 2017). Written informed consent was obtained from each participating doctors in this study.

### Sampling methods and participants

**Fig. 1:**
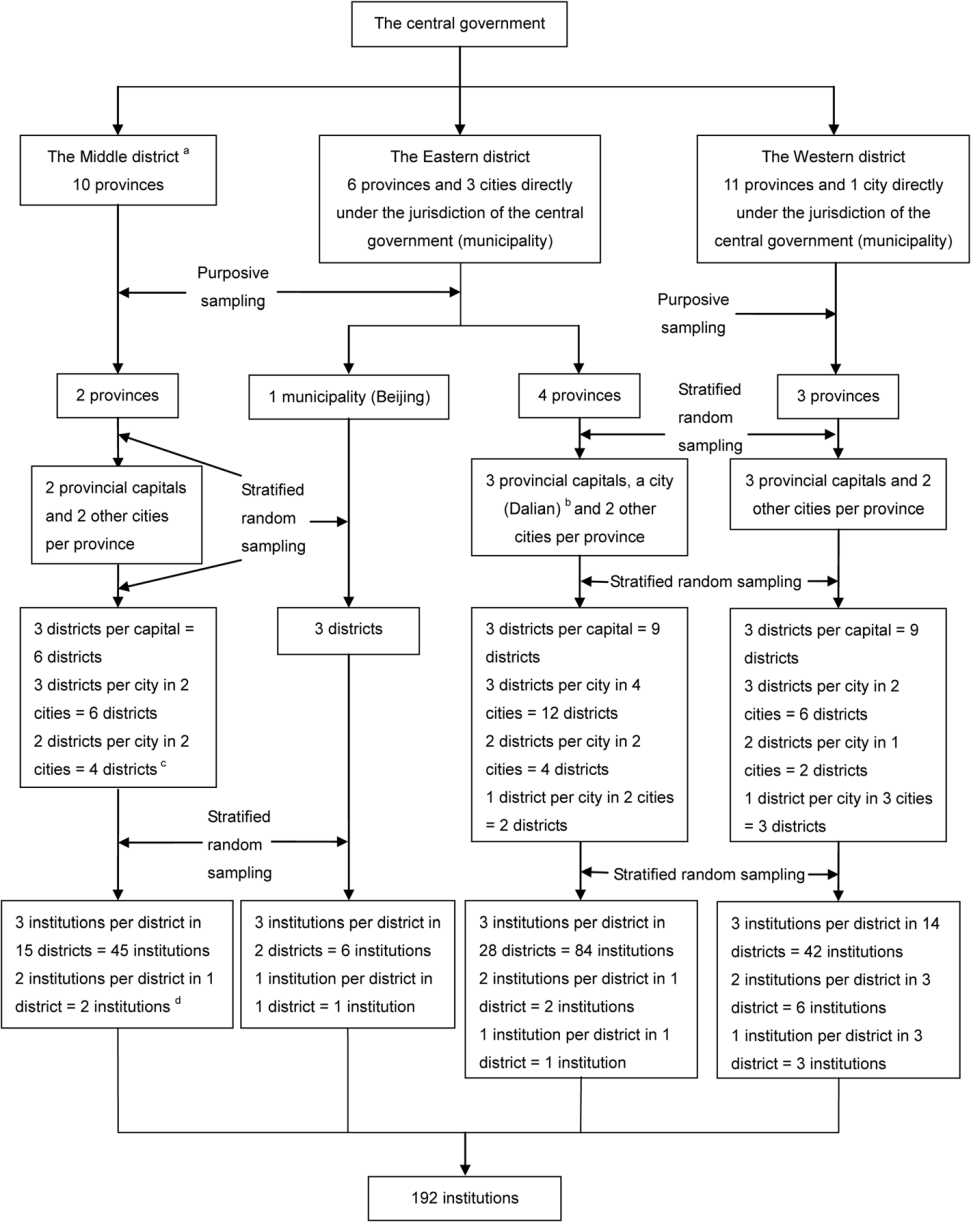
Sampling flow of CHSIs in urban China in 2011. Note: ^a^ The central district includes 10 provinces (Hebei^*^, *Shanxi*, *Jilin*, *Heilongjiang*, *Anhui*, *Jiangxi*, *Henan*, Hunan^*^, Hubei and *Hainan*). The eastern district includes six provinces (Liaoning^*^, Shandong^*^, *Jiangsu*, Zhejiang^*^, Fujian^*^ and *Guangdong*) and three cities that are directly under the jurisdiction of the central government (Beijing^*^, *Shanghai* and *Tianjin*). The western district includes 11 provinces (*Inner Mongolia*, Guangxi^*^, *Sichuan*, Guizhou^*^, *Yunnan*, *Xizang*, *Shanxi*, *Gansu*, *Qinghai*, Ningxia^*^, and *Xinjiang*) and one city that is directly under the jurisdiction of the central government (*Chongqing*). ^*^ Provinces selected for the study. ^b^ *Dalian* is one of the five cities (*Xiamen*, *Ningbo*, *Qingdao*, *Shenzhen*, and *Dalian*) that are specifically designated in the state plan and similar in size and economic situation to a provincial capital. ^c^ The plan was to examine three districts per city, though some of the selected cities had fewer than three districts. ^d^ The plan was to sample three institutions per district per city, though some selected districts had fewer than three institutions

The general stratified sampling method was adopted to select 9 provinces and one municipality from the available 31 provinces (including five autonomous regions and four municipalities) which divided into three regions (eastern region, central region and western region) in Mainland China;then the provincial capital cities and two other random cities (from above and below the median GD) were selected from each province; then 3 city districts based on good, moderate and poor economic condition were selected from each city; finally, 3 CHSIs were selected according to the CHSIs’ development status (good, moderate or poor). In summary, 9 provinces and one municipality, 28 cities, 66 districts and 192 CHCs were selected for the survey. (the sampling flow was described in Fig. 1) We investigated all doctors who were regular employees at 192 CHSIs, rehired retirees and temporary employees were excluded. Overall, 3098 eligible doctors in the selected CHSIs were sent questionnaires to complete and return.

### Data collection and quality control

The investigation studied the training status and needs of doctors in Chinese urban CHSIs. Fieldwork was performed from May to November 2011. The quality assurance measures for this investigation included evaluating the questionnaire, training the investigators, and asking a fieldwork supervisor to monitor the investigation process. Before the investigation was implemented, it was reviewed, edited, and validated by 29 experts from health administrative department, clinical teaching hospital and CHSIs. The questionnaire (see Additional file 1) consists of three parts of contents as following: characteristic of health providers (e.g. socio-demographic, workplace, educational and title level), training status (e.g. the types of training programs, training contents, and training problems), and training needs (e.g. the attitude to training, training motivation, the knowledge and skills needs of training, etc). Collective training for 126 coordinators from investigated provinces was held in Beijing on May 2, 2011 by Technology and Education of China’s Ministry of Health and our research group. They implemented the ensuing training for investigators at community-level in the next stage. Three hundred and eighty four administrative leaders of the CHSIs and fieldwork supervisor were responsible for monitoring the process. They checked the completed questionnaires to avoid missing data. A pilot survey of 100 participants was conducted between May 6 and May 10, 2011, in Beijing to assess the feasibility of the questionnaire and the fieldwork procedures. The questionnaires and data were validated by our research experts. All data adopted double entry by using EpiData software (Version 3.1, EpiData association, Odense, Denmark). The two databases were compared and analyzed for discrepancies. If discrepancies exist, the original data source would be reviewed. To avoid wrong information disturbing the research results, logical contradiction and missing data of items in the questionnaires which we will analysis in the manuscript were confirmed via telephone to correct and supplement information.

### Statistical analysis

All analyzes were conducted by using the Statistical Package for Social Sciences (SPSS) for Windows (Version 17.0; SPSS, Inc., Chicago, IL, USA) Descriptive statistics (frequencies and percentages) were used to describe the characteristics of doctors status and needs of training. Differences of training contents, problems and needs between eastern, central and western regions were tested with chi-square tests. The tests was two-sided, and *P* < 0.05 was considered statistically significant.

## Results

### Characteristics of doctors

A total of 3037 (out of 3098) doctors consented to participate in this study and the remaining 61 declined, and the response rate was 98.0%. Table 1 shows the social demography characteristic of participants in three regions. In all, 39% were male and 61% were female. There was significant difference in education level (*P* < 0.001) and title level (*P* < 0.001) of doctors among three regions. In three regions, the doctors of western region had the highest education level. Both doctors of the central region had the lowest education level in three regions. In three regions, more doctors of western region (119, 16.2%) had the senior title level than eastern region (214, 12.1%) and central region (59, 10.9%), while doctors of central region had the lowest title level.

**Table 1:** Distribution of total participants by the social demography characteristics in three regions

	Doctors (NO.%)				*P*
	Eastern	Central	Western	Total	
CHSIs Number	94(48.9)	47(24.5)	51(26.6)	192(100)	
Gender					0.195
Male	684(38.8)	212(39.3)	288(39.2)	1184(39.0)	
Female	1078(61.2)	328(60.7)	447(60.8)	1854(61.0)	
Education level*					*< 0.001*
Undergraduate and above	837(47.5)	229(42.4)	434(59.0)	1500(49.3)	
Junior college	593(33.7)	236(43.7)	196(26.7)	1025(33.8)	
Technical secondary school/ High school	313(17.8)	73(13.5)	105(14.3)	491(16.2)	
Below high school	19(1.1)	2(0.4)	0(0.0)	21(0.7)	
Title level#					*< 0.001*
Senior title	214(12.1)	59(10.9)	119(16.2)	392(12.9)	
Middle title	657(37.3)	152(28.1)	277(37.7)	1086(35.8)	
Junior title and below	786(44.6)	275(50.9)	291(39.6)	1352(44.5)	
No title	105(6.0)	54(10.0)	48(6.5)	207(6.8)	

Note: SD = standard deviation

* In China, medical educational programs include 5- to 8-year post–high school training programs, 3-year post–high school programs which are decreasing, 4-year post–middle school programs which have almost disappeared, and barefoot doctors (who are basically farmers with no proper medical education, usually only 3 to 6 months of basic medical training, and take care of the primary health care needs in their communes)

# Senior title (assistant chief physician or chief physician)

Middle title (attending physician)

Junior title and below (resident physician or assistant doctor)

**Fig. 2:**
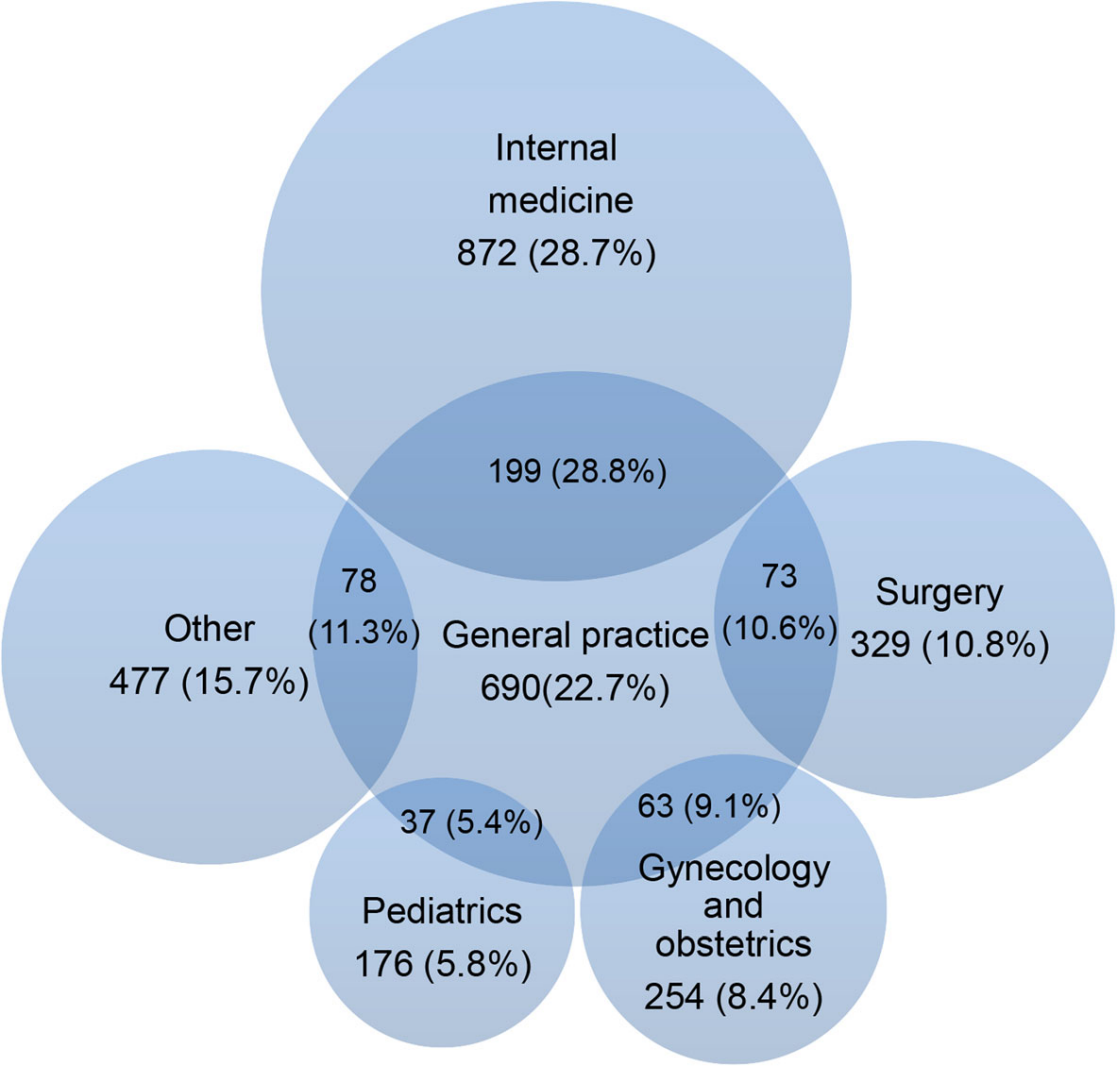
The proportion of doctors registered in physician practice scope of clinical categories. Note: The Chinese government has ruled that doctors in primary health care institutions can register no more than three different specialties in one category. Source: National Health and Family Planning Commission of the People’s Republic of China. The notice of interim provisions on physicians practicing registered scope issued by Ministry of Health. Available from: http://www.gov.cn/gongbao/content/2002/content_61429.htm

Figure 2 shows that the top five categories of physicians’ registration reported were internal medicine (872, 28.7%), GP (690, 22.7%), surgery (329, 10.8%), gynecology and obstetrics (254, 8.4%) and pediatrics (176, 5.8%).

### Training status

**Fig. 3:**
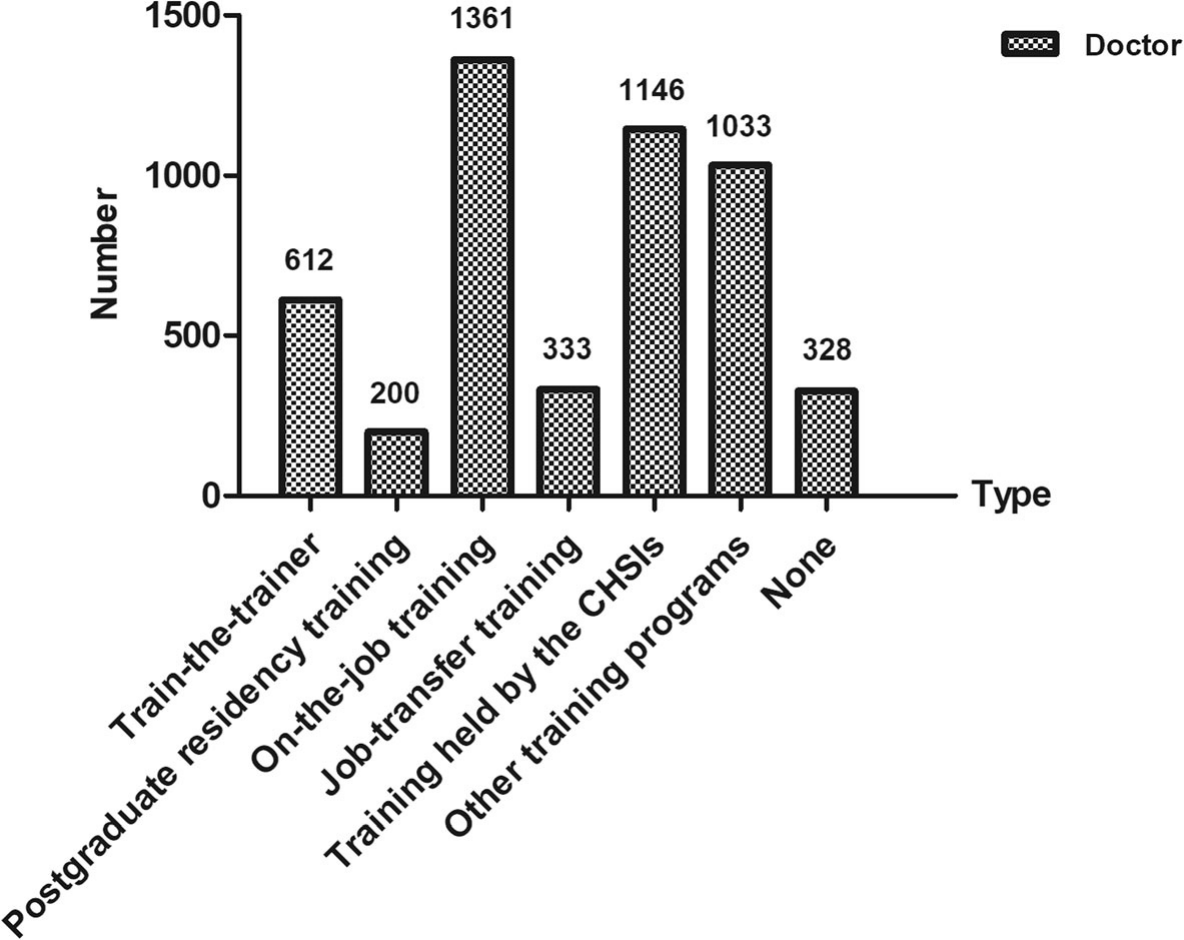
The types of training programs attended by the CHSIs doctors. Note: Doctors may attend more than one type of training, and some values overlapped

Figure 3 indicates that 89.2% (2709) doctors had attended at least one type of training program. 1361 (44.8%) and 333 (11.0%) had attended on-the-job training and job-transfer training programs respectively. 612 (20.2%) had attended the train-the-trainer program. Only 200 (6.9%) doctors had attended postgraduate residency training program. Table 2 indicates the differences between a series of training programs for GPs in China.

**Table 2:** The differences between a series of training programs for GPs in China

Differences	Postgraduate residence training program	On-the-job training program/job-transfer training program	Train-the-trainer training program	Training program held by CHSIs
Target population	Graduates whose medical background is 5- to 8-year post–high school education or over, and who are likely to choose general practice as a career.	Community health physicians with 3- to 5-year post–high school medical education, who are willing to become general practitioners by on-the-job training.	Clinical faculty from hospital, clinical faculty from CHSIs and theoretical faculty from university	Doctors working in CHSIs.
Teaching contents/curriculum	(1) Hospital-based Clinical rotation; (2) CHSIs-based training.	(1) Academic curriculum; (2) Hospital-based clinical rotation; (3) CHSIs-based training.	(1) Task responsibilities and contents for GP faculty; (2) Clinical teaching contents and methods; (3) Theoretical knowledge and clinical skill;	Basic medical services, chronic management, preventive care, etc.
Training lengths	33 months (full-time)	12 months (full-time)	2 months	Irregularly scheduled
Type of certificates obtained	Standardized residency training certificate which is issued by Ministry of Health and is a prerequisite for the qualification of GP.	GP on-the-job training certificate which is issued by provincial health department.	GP faculty training certificate which is issued by provincial health department.	None

Table 3 shows the types of training programs attended by the CHSIs doctors in three regions. Compared with central and eastern regions, fewer doctors in western region had received on-the-job training. The doctors of central region had lowest ratio in participating postgraduate residency training.

**Table 3:** The types of training programs attended by doctors in CHSIs in three regions

Type of training program	Doctor (NO.%)			*P*
	Eastern	Central	Western	
On-the-job training	869(49.3)	232(43.0)	260(35.4)	*< 0.001*
Postgraduate residency training	137(7.8)	13(2.4)	50(6.8)	*< 0.001*
Job-transfer training	170(9.7)	56(15.9)	77(10.5)	*< 0.001*
Train-the-trainer	361(20.5)	118(21.9)	133(18.1)	0.349
Training held by the CHSIs	776(44.0)	161(29.8)	209(28.4)	*< 0.001*

Note: Doctors may attend more than one type of training, and some values overlapped

Table 4 describes the topics and problems in the training programs reported by doctors in CHSIs in three regions. Comparing with eastern and central regions, the doctors of western region received least training in *“community health service competency”* and *“clinical practice skills”*. The biggest problem in the training programs for doctors in all three regions was *“insufficient training time”*.

**Table 4:** The training contents, problems and needs of doctors in CHSIs in three regions

	Doctor (NO.%)			*P*
	Eastern	Central	Western	
Training topics				
Basic clinical theory knowledge	880(52.4)	272(50.4)	333(45.3)	0.081
Clinical practice skills	898(51.0)	282(52.2)	337(45.9)	*0.034*
Community health service competency	908(51.5)	322(59.6)	314(42.7)	*< 0.001*
Preventive care	364(20.7)	128(23.7)	145(19.7)	0.200
Training problems				
An excessively short training time	649(36.8)	197(36.5)	291(39.6)	0.379
Insufficient training content	575(32.6)	181(33.5)	219(29.8)	0.284
Deficiency in clinical practice skills	451(25.6)	156(28.9)	182(24.8)	0.215
Insufficient resolution of problems at work	465(26.4)	143(26.5)	182(24.8)	0.674
Knowledge needs				
The updated international medical knowledge	1406(79.8)	427(79.1)	532(72.4)	0.066
The updated domestic medical knowledge	1357(77.0)	422(78.1)	538(73.2)	*< 0.001*
Clinical experience	549(31.2)	201(37.2)	206(28.0)	*0.002*
Clinical decision making	488(27.7)	164(30.4)	183(24.9)	0.092
Clinical medication	462(26.2)	162(30.0)	195(26.5)	0.213
Skill needs				
Communication skills	1481(84.1)	446(82.6)	569(77.4)	*< 0.001*
Diagnosis and differential diagnosis	1446(82.1)	442(81.9)	575(78.2)	0.074
Physical examination	1385(77.1)	407(75.4)	509(69.3)	*< 0.001*
Observation skills	1335(75.8)	406(75.2)	519(70.6)	*0.024*
Clinical medication use	1317(74.7)	401(74.3)	502(68.3)	*0.003*

### Training needs

Most of the doctors (95.0%) were willing to attend training programs regularly. Table 4 also describes the top five knowledge and skill training needs of doctors. The doctors of eastern and central regions had more knowledge needs of *“the updated domestic medical knowledge”* and “*The updated international medical knowledge*” than western region. Comparing to the western region, doctors of the eastern and central regions had more skill training needs of *“communication skills”*, *“physical examination”*, *“observation skills”* and *“clinical medication use”*.

## Discussion

The recent House of Commons Health Select Committee report on the workforce argued that “the quality, safety, effectiveness and efficiency of health care services depend on the availability of sufficient numbers of well-trained and well-motivated staff” [12]. At present, there are many challenges facing general practice, including rising patient and physician expectations, growing comprehensive and continuous of care, burgeoning technique, and expanding knowledge base [13]. Thus, it is necessary to increase more well-trained GPs to play the role of gate-keeper in CHSIs of China. In our study, the participants have high willingness to receive training. Furthermore, 89.2% doctors had attended at least one type of training program, including on-the-job training, job-transfer, and other CME programs, etc.

From the international perspective, the scopes and contents of training programs need to be designed and broadened to meet the knowledge and skill needs for doctors. There is a trend toward competency-based rather than time-based training, with the trainees expected to demonstrate certain competencies to enhance the level of training [14, 15]. Six core competencies relevant to any modern domain of medical practice should be emphasized on training programs, including patient care, medical knowledge, communication skills, professionalism, practice-based learning and improvement, and system-based practice [16]. At present, the training contents for GPs concentrate on customized contents of doctor-patients communication skills, community-based healthcare competency, clinical skills and knowledge [17–19]. Such as the “Practice Support Program of Canada”, topic areas range from clinical tools/skills to office management, and the five learning modules consist of advanced access, chronic disease management, patient self management, group medical visits, and adult mental health were trained in three half-day learning sessions and 6–8 week practice periods [20]. Compared with the training contents of other country, our findings demonstrated that the training contents in three regions, were similar with the competency-based training contents. Nevertheless, we also found some key problems, including insufficient training time, deficiency in clinical practice skills, and lack of practical training contents. Actually, limited understanding of the clinical decision-making processes and difficulty in translating knowledge to practice become common phenomenon [21]. So it is necessary to highlight training contents with strong practicability in training programs at present and in the future. It is also suggested that teaching hospitals should train GPs to grasp the basic skills such as the film reading of X-ray, CT, MRI and ECG, emergency treatment, and physical examination in clinical rotations [5]. Insufficient training time is a worldwide problem, which has been attributed to increasing workloads and far distance from training place [22]. For doctors, flexibility of training time is recommended as an effective method to resolve the contradiction between work and training, so training in CHSIs themselves would attract more trainees. The work-hour constraints requires training model to find the proper balance between continuity of care and the trainee fatigue. Worldwide, many configurations and approaches can compensate for this obstacle, including videoconferencing, distance learning, and web based learning [23–25]. It is worth mentioning that the “communication skills” was the most important skill for doctors in this study. The role of patients’ support and gratitude could reduce health care providers’ burnout [26]. Professionalism and interpersonal communication skills have been incorporated formally into medical training recently. Knowing when, what, and how to communicate with patients can be seen as an essential part of skills training. Teaching hand in hand, [27] providing a “toolkit” of skill, [28] and personal reflection [27] could be applied to overcome the problems in communication skill training.

In general, doctors in three regions had similar training courses, problems and needs. While, doctors of western region receive least contents related to “community health service competency”, and had least knowledge and skill needs than other two regions. Comparing with the advantage of economic development for eastern region, the central and western regions have financial and medical care policy support provided by central government to develop community health services and general practice training, especially in western region [29]. In Ningxia, most of the CHSIs were transformed from secondary or tertiary hospitals, while CHSIs in other districts were independently regulatory institutions, thus health care providers especially doctors needed to meet more restrict criteria to enter the hospitals. In the future, “community health service competency” including chronic disease management, preventive care should be considered as the important parts of training in western regions.

### Strengths and limitations

The strengths of this study are that it has brought questionnaire investigation on the current training programs and training needs of doctors in CHSIs in nationally. We carried out a series of quality control measures by a multidisciplinary team to ensure a depth of understanding critical to the design of the study and the validity of results. However, several limitations need to be considered: (1) although the study selected different CHSIs from the eastern, central and western regions to decrease the likelihood of bias, the use of purposive sampling restricted the generalizability of results because more of the relatively developed districts in the central and western regions were investigated. (2) although there were no sensitive topics, and each participant wrote informed consent, it is undeniable that participants which were not anonymised to answer the questionnaires will reduce the authenticity of research to some extent; (3) we mainly investigated the subjective opinions of the participants rather than objective indicators. Thus, the evaluation that focuses on structure and process should be implemented to discover the problems of training programs. In further research, the study will focus on the periodic evaluation of their knowledge and competency in career; (4) although the research took place in 2011, until now, there was no similar nationwide survey conducted in China. In recent years, the Chinese government has gradually realized the importance of primary care and issued some relevant policies and regulations. In 2018, the Chinese government proposed a comprehensive reform to improve the training of GPs, and some measures will be taken to promote the training programs. The results of our study could provide propositions for training more qualified GPs in the future.

## Conclusions

China is suffering from a severe shortage of qualified GPs. Under the work-hour restriction circumstance, government should learn the advanced international education and training models to improve the effectiveness of training programs. Moreover, the government should design proper training contents according to the knowledge and skill needs of the different regions. Furthermore, a uniform, rigorous training and evaluation system focus on practicability should be established to promote community health service system in Mainland China.

## Additional file

Additional file 1: An questionnaire investigation of training status and needs for doctors working in community health service institutions in China. Note: The questionnaire was designed to collect some information about the training status and needs for doctors working in community health service institutions in China. (DOC 58 kb)

## Abbreviations

CHSIs: Community health service institutions
CME: Continuing medical education
GPs: General practitioners
SPSS: Statistical Package for Social Sciences
WONCA: World Organization of Family Doctors
