# Supplementary material for: The training contents, problems and needs of doctors in urban community health service institutions in China
Source: BMC Fam Pract. 2018 Nov 28;19:182. doi: 10.1186/s12875-018-0867-6 (PMC6263560; doi:10.1186/s12875-018-0867-6)
Supplement: Supplementary file 1 — An questionnaire investigation of training status and needs for doctors working in community health service institutions in China. Note: The questionnaire was designed to collect some information about the training status and needs for doctors working in community health service institutions in China. (DOC 58 kb) [file 12875_2018_867_MOESM1_ESM.doc]

**Additional file 1**

**An questionnaire investigation of training status and needs for doctors working in community health service institutions in China**

Number ______________

Province/Municipality directly under the central government __________

City/District/State _______________

District _______________

Health service institution ______________

Investigate date: _____________ Signature of investigator:______________

Check date: _____________ Signature of scrutator: _______________

**Dear doctors:**

The research is part of the Nationwide Investigation on Health Professionals of Community Health Service Institutions in Urban China and sponsored by the Department of Medical Science, Technology and Education in China’s Ministry of Health. The questionnaire is only used to investigate your training status and needs. Your thoughts are important to improve the training program. Please take a few minutes to fill in the following questions carefully. We assure you that your answers will be kept strictly confidential.

Thank you for your support.

National community health service research team

Note:

1. Doctors working in the community health service institution are included.

2. Rehired retirees and temporary employees are excluded.

(Please mark the “√” under the numbers, e.g. ①, ②, or write the content on the “_____” following the request of each question)

**Part 1. Social demography characteristic**

1. Name: ________ Telephone number: ____________

2. Gender: ① Male ② Female

3. Birth date: Year ________ Month________

4. Educate level

①Undergraduate and above

②Junior college

③[Technical secondary school](javascript:;)/ High school

④Below high school

5. What is the type of practicing qualification you acquired?

①Practicing physician

②Practicing assistant physician

③None (Please answer the question 7)

6. Categories of physicians’ registration（Multiple choices）

①General practice

②Internal medicine

③Surgery

④[Gynecology and obstetrics](javascript:void(0);)

⑤Pediatrics

⑥Orthopsychiatry

⑦Rehabilitation medicine

⑧Chinese traditional medicine

⑨Other ______________________

7. What is the highest title level you acquired?

①Senior title (assistant chief physician or chief physician)

②Middle title ([attending](http://cn.bing.com/dict/search?q=Attending&FORM=BDVSP6&mkt=zh-cn) [physician](http://cn.bing.com/dict/search?q=physician&FORM=BDVSP6&mkt=zh-cn))

③Junior title and below ([resident](http://cn.bing.com/dict/search?q=resident&FORM=BDVSP6&mkt=zh-cn) [physician](http://cn.bing.com/dict/search?q=physician&FORM=BDVSP6&mkt=zh-cn) or assistant doctor)

④None

8. Where is your main workplace?

①Community health service center

②Community health service station

③Other _____________________

**Part 2. Training status**

9. What is (are) the type(s) of training program(s) you attended？（Multiple choices）

①None (Please answer the question 14)

②Train-the-trainer

③Postgraduate residency training

④On-the-job training

⑤Job-transfer training

⑥Training held by the CHSIs

⑦Other______________________

10. What is (are) the topic(s) of the training program(s)? （Multiple choices）

①Basic clinical theory knowledge

②Clinical practice skills

③Community health service competency

④Preventive care

⑤Other ________________________

11. What is (are) your purpose(s) of attending training programs? （Multiple choices）

①Improve service skill

②Enhance theoretical level

③Study new knowledge

④Resolve problems in work

⑤Acquire the qualification

⑥Other___­­­­­________________

12. What is (are) the main problem(s) in the training programs? （Multiple choices）

①An excessively short training time

②An excessively long training time

③Insufficient training content

④Deficiency in clinical practice skills

⑤Insufficient resolution of problems at work

⑥Insufficient training materials

⑦Repetition contents

⑧Insufficient updated knowledge

⑨Other___­­­­­________________

13．Do you satisfied with the training programs you attended?

①Yes

②No

③I don't know

**Part 3. Training needs**

14. Do you want to attend the training programs?

①Yes

②No

③I don't know

15. What is (are) the knowledge do you want to learn from the training programs in the future? （Multiple choices）

①The updated international medical knowledge

②The updated domestic medical knowledge

③Clinical experience

④Clinical decision making

⑤Clinical medication

⑥Communication skills

⑦Other__________________

16. What is (are) the skill(s) do you want to learn from the training programs in the future? （Multiple choices）

①Communication skills

②Diagnosis and differential diagnosis

③Physical examination

④Observation skills

⑤Clinical medication use

⑥Medical record writing

⑦Health education

⑧Community diagnosis

⑨Other__________________

Thank you very much for taking the time to complete this survey. Your feedback is valued and very much appreciated.
